# Fires in the Cenozoic: a late flowering of flammable ecosystems

**DOI:** 10.3389/fpls.2014.00749

**Published:** 2015-01-05

**Authors:** William J. Bond

**Affiliations:** South African Environmental Observation Network – National Research Foundation and Department of Biological Sciences – University of Cape TownRondebosch, South Africa

**Keywords:** savannas, shrubland, flammable ecosystems, fossil charcoal, phylogenetic habitat reconstruction

## Abstract

Modern flammable ecosystems include tropical and subtropical savannas, steppe grasslands, boreal forests, and temperate sclerophyll shrublands. Despite the apparent fiery nature of much contemporary vegetation, terrestrial fossil evidence would suggest we live in a time of low fire activity relative to the deep past. The inertinite content of coal, fossil charcoal, is strikingly low from the Eocene to the Pleistocene and no charcoalified mesofossils have been reported for the Cenozoic. Marine cores have been analyzed for charcoal in the North Pacific, the north and south Atlantic off Africa, and the south China sea. These tell a different story with the oldest records indicating low levels of fire activity from the Eocene but a surge of fire from the late Miocene (~7 Ma). Phylogenetic studies of woody plants adapted to frequent savanna fires show them beginning to appear from the Late Miocene with peak origins in the late Pliocene in both South American and African lineages. Phylogenetic studies indicate ancient origins (60 Ma+) for clades characteristic of flammable sclerophyll vegetation from Australia and the Cape region of South Africa. However, as for savannas, there was a surge of speciation from the Late Miocene associated with the retreat of closed fire-intolerant forests. The wide geographic spread of increased fire activity in the last few million years suggests a global cause. However, none of the potential global factors (oxygen, rainfall seasonality, CO_2,_ novel flammable growth forms) provides an adequate explanation as yet. The global patterns and processes of fire and flammable vegetation in the Cenozoic, especially since the Late Miocene, deserve much more attention to better understand fire in the earth system.

Fire shapes the dominant growth forms in a large proportion of the world’s ecosystems. In the tropics and subtropics, savannas occur over vast areas accounting for more than half the world’s annual burnt area (van der Werf et al., 2010). At high latitudes, boreal forests cover nearly a fifth of the vegetative land surface of the world and burn regularly. Flammable shrublands are most apparent in Mediterranean climate regions but also occur elsewhere especially where the climate is unsuitable for C4 grasses. The southern two thirds of Australia, for example, are dominated by flammable shrublands often with emergent eucalypt trees. In all these regions closed forests that resist burning occur in a bi-stable state with the shrublands suggesting that climate alone does not explain vegetation structure. The dawning recognition that fire is a major factor shaping global biome distribution has emerged in little over a decade from diverse studies (e.g., Bond et al., 2005; Bowman et al., 2009; Krawchuk et al., 2009).

The world was not always this fiery. Fire activity has waxed and waned throughout the long history of terrestrial vegetation (Scott, 2000; Pausas and Keeley, 2009; Glasspool and Scott, 2010; Belcher et al., 2013). Fossil evidence indicates that fires were particularly common in the Carboniferous and Permian (Falcon-Lang, 2000; Uhl and Kerp, 2003; Jasper et al., 2013). In the Cretaceous, fires were again common and may have promoted the spread of angiosperms at the expense of ancient gymnosperms (Bond and Scott, 2010; Brown et al., 2012; He et al., 2012). These periods of high fire activity have been linked to above ambient concentrations of oxygen in the atmosphere (Scott and Glasspool, 2006; Belcher and McElwain, 2008; Belcher et al., 2010). Yet there is a fundamental conundrum in the geological record of fire. Although high fire activity in the Cretaceous continued into the Paleocene (Collinson et al., 2007; Belcher et al., 2013) *fossil evidence for fire activity over the last 50*+ *million years (Ma) from the Eocene through to the present day, is scant* (Scott, 2000). Indeed the record of inertinite, the charcoal content of coal and a major proxy for ancient fire activity, shows the lowest fire activity in almost the entire geological record in Cenozoic coals (**Figure 1**).

**FIGURE 1:**
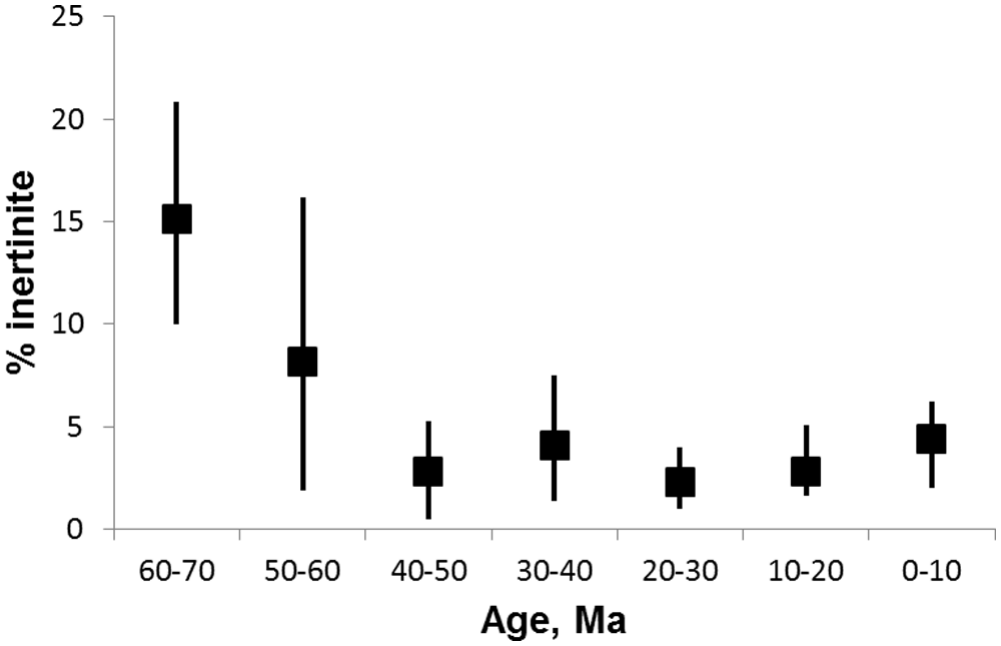
**Inertinite abundance from the Late Cretaceous through the Cenozoic (65 to 0 Ma).** Inertinite is the fossil charcoal content of coal. Data from Glasspool and Scott (2010), Supplementary Table S2. The median (squares) and upper and lower quartile values for each age bin are shown.

The conundrum is that we live in what seems to be a very flammable world where fire is influential in structuring global vegetation. Yet the inertinite record suggests we are living in a world of very low fire activity. Is the current extent of pyrophytic vegetation the norm for the last 50+ million years implying that, relative to the Cretaceous or Permian, we are living in a low fire world? Would we have a different picture from using different proxies for past fires? Was there a late Cenozoic surge in fire activity that produced our modern pyrophytic biomes? If fires did increase, worldwide, in the late Cenozoic what caused this increase? And finally, what are the implications of Cenozoic fire history for interpreting ancient fires in the Mesozoic and Paleozoic? In this paper, I review evidence for fire in the Cenozoic (the last 65 Ma) focusing particularly on the Neogene (23–2.6 Ma) when modern ecosystems emerged.

## TYPES OF EVIDENCE FOR PALEOFIRES

### FOSSILS

Scott (2010) has reviewed proxies for fire activity in the fossil record. One of the most useful proxies is the inertinite content of coal since there is a near continuous record in coal deposits from ~420 Ma (Diessel, 2010; Glasspool and Scott, 2010). The Cenozoic record of inertinite (**Figure 1**) indicates high fire activity in the Paleocene (65–55 Ma), a steep decline in the Eocene (55–34 Ma) and low activity right through to the present (Diessel, 2010; Glasspool and Scott, 2010). Shearer et al. (1995) suggested that coal properties, including inertinite, changed fundamentally in the Cenozoic as a result of the switch from gymnosperm to angiosperm dominance in ecosystems with the former having a much higher lignin content preserved in coal. The rise of the angiosperms and increasing herbaceous material from the Mesozoic to the Cenozoic therefore suggests one possible explanation for the poor fossil record of fire in the latter. Charcoalified flowers in the Cretaceous are a rich source of data on early angiosperm evolution (Friis and Skarby, 1981; Friis et al., 2011) and also a marker for higher fire activity from the mid-Cretaceous coincident with angiosperm spread (Bond and Scott, 2010; Brown et al., 2012). Charcoalified plant organs have not been reported for the Cenozoic (Scott et al., 2013). However, charcoalified plant organs are meso-fossils requiring special methods of preparation (Schönenberger, 2005) and may simply have been overlooked in Cenozoic studies.

Charcoal is the most widely used proxy for fire activity in Quaternary studies but there are many problems in inferring ancient fire activity from charcoal (see e.g., Keeley et al., 2012; Belcher et al., 2013). These include intrinsic taphonomic biases against finding flammable ecosystems. For example, Keeley et al. (2012) noted the absence of *Adenostoma fasciculatum*, the most common shrub in Californian chaparral, in the fossil record. Fire-resistant gallery forests, common in fire-prone grassy systems, filtered the charcoal record entering a lake from adjacent frequently burning African savannas (Aleman et al., 2013). Inertinite develops in peatlands which are uncommon in seasonally arid savannas and Mediterranean shrublands such as chaparral (Keeley et al., 2012). Woody vegetation produces more charcoal in larger fragments than grasslands so that charcoal evidence for the spread of tree-less grasslands is likely to be grossly underestimated. Furthermore, the deep weathering characteristic of oxisols on ancient peneplains in tropical landscapes biases against fossil formation in humid tropical climates. Thus savanna fossils are likely to be biased to more arid areas where low plant productivity would limit fires.

Marine charcoal observed in cores from ocean drilling avoids some of these problems. Grass cuticles have been recovered from such cores presumably lofted into the ocean from smoke plumes (Herring, 1985; Morley and Richards, 1993). However, it is difficult to discern the source of the charcoal and the extent to which paleowinds have redistributed it. Interpretation of the vegetation source of the charcoal is also confounded by uncertainty over fluvial or aeolian origins of black carbon. Fossil sites are also rare in many parts of the world and there is an understandable tendency for paleoecologists to extrapolate over large geographic regions from rare point samples.

### PHYLOGENIES

Phylogenetic methods have recently been applied as additional tools to explore the history of pyrophytic vegetation (e.g., Simon et al., 2009; Bytebier et al., 2011; He et al., 2011, 2012). Dating is based on molecular clock assumptions calibrated with fossils of known age. The many difficulties in developing dated phylogenies have been met with increasingly sophisticated analytical models and, at least among systematists, there is growing confidence in the accuracy of molecular dating (Smith et al., 2010). Disagreement between molecular dating and fossil evidence is particularly glaring for groups with a well-known fossil record such as mammals (e.g., Foote et al., 1999) but even for the mammals fossil and molecular clock dates are beginning to converge with more refined phylogenetic and dating methods (e.g., Bibi, 2013). But there are additional problems in using phylogenetic methods for exploring ecological questions. A key question for this paper is when novel growth forms, such as C4 grasses, assembled into ecosystems that began to alter their environment by burning on a regular basis. Phylogenies are based on origination and diversification and it is not clear how diversification processes relate to increased ecological extent or importance of an evolutionary innovation. Sometimes the evolution of a new growth form may produce a novel biotic habitat which may then initiate novel radiations of species dependent on that habitat. For example, epiphytic ferns began to diversify in the early Cenozoic when, presumably, more trees became available as substrates. Phylogenetic dating places their diversification in the Eocene (from 55 Ma) consistent with fossil evidence for widespread emergence of angiosperm-dominated forests (Schuettpelz and Pryer, 2009). But the opposite pattern is also possible. Diversification rates may decline as ecological dominance of a lineage increases. An evolutionary innovation which generates new habitat would initially be distributed in small isolated patches. If the innovation is successful, fragmented habitats may coalesce resulting in a reduction in speciation rates as barriers to gene flow fall away. Bouchenak-Khelladi et al. (2014), for example, found no phylogenetic signal coincident with the rise of the savanna biome in a large grass phylogeny. Linder (2008) has provided a thoughtful discussion of phylogenetic signals associated with diversification. Exposure to new climates and soil types, such as might occur if flammable grasslands rolled back fire-sensitive forests, would be expected to generate speciation. But where such forests occurred in similar climates and soils, the expansion of flammable communities may merely lead to a re-shuﬄing of existing taxa rather than speciation (e.g., Ackerly, 2004).

Here I explore both fossil and phylogenetic evidence for fire activity linked to the development of pyrophytic biomes. I first consider savannas and related biomes of the tropics and subtropics where coincident increased fire activity has been linked to the enigmatic late Miocene (11.5–5.3 Ma) appearance of this major biome (Bond et al., 2003a; Keeley and Rundel, 2005; Osborne, 2008, 2011). I then consider evidence for fire activity in pyrophytic woody vegetation, focusing on Mediterranean type shrublands and open woody vegetation of Australia and the Cape region of South Africa. Finally I consider possible causes of a global increase in fire activity which might explain the rise of pyrophytic biomes in the last ~10 Ma.

The review is restricted to savannas and temperate flammable shrublands and associated woodlands since they have been the focus of much recent research on the evolution and history of flammable ecosystems, especially those dominated by angiosperms. I have not considered the sparse literature on the Cenozoic history of flammable boreal ecosystems complicated by glaciation in the Pleistocene. The role of fire in the emergence of steppe grasslands, dominated by C3 grasses, would be an interesting contrast to savannas but I was unable to find studies on the subject.

## FIRE IN THE TROPICS AND SUBTROPICS: THE RISE OF SAVANNAS

### FOSSIL CHARCOAL

**FIGURE 2:**
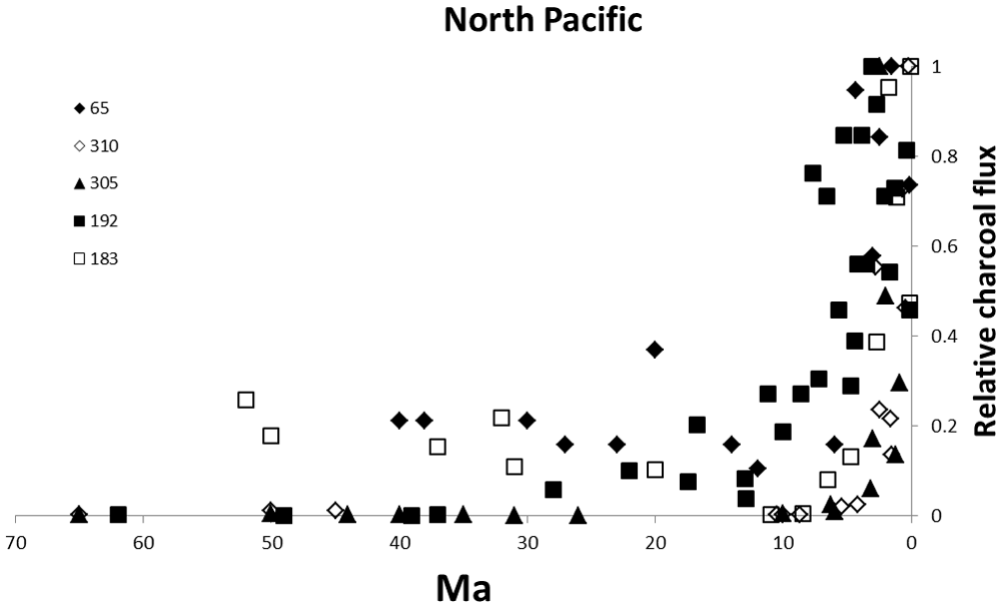
**Cenozoic charcoal flux from ocean cores in the North Pacific.** Data from Herring (1985). The legend numbers refer to localities. Latitudes N, 65 4°, 310 36°, 305 32°, 192 53°, and 183 52°. Values are relativized to the maximum charcoal recorded at each site.

There has been intense interest in the origin of grasslands generally and of C4 grass dominated savannas in particular (Cerling et al., 1997; Jacobs et al., 1999; Keeley and Rundel, 2005; Osborne, 2008; Edwards et al., 2010; Strömberg, 2011). Osborne (2008) reviewed the multiple hypotheses proposed for the spread of the savanna biome. The most widely cited evidence for an association between the rise of the savanna biome and fire comes from charcoal records from the Deep Sea Drilling Project in the North Pacific (Herring, 1985; Bond et al., 2003a; Keeley and Rundel, 2005; Beerling and Osborne, 2006; Osborne, 2008). Herring (1985) analyzed charcoal from eleven cores spanning a latitudinal range from near the equator to 53°N and a temporal range from Late Cretaceous to the Quaternary. The older sediments showed low, but measurable, charcoal fluxes (mass of charcoal per unit area per unit time) for the early Cenozoic (from ~65 Ma in some cores) with generally low charcoal fluxes from then until the late Miocene. There was a sharp increase in charcoal between 10 and 1 Ma (**Figure 2**). Low latitude cores show a surge in charcoal fluxes from the late Miocene (~7 Ma) or younger coinciding with the expansion of C4 savannas as recorded in the carbon isotopic record (Cerling et al., 1997). Though Herring reported the presence of charred grass cuticles, it was generally not possible to identify the biological source of the charcoal.

**FIGURE 3:**
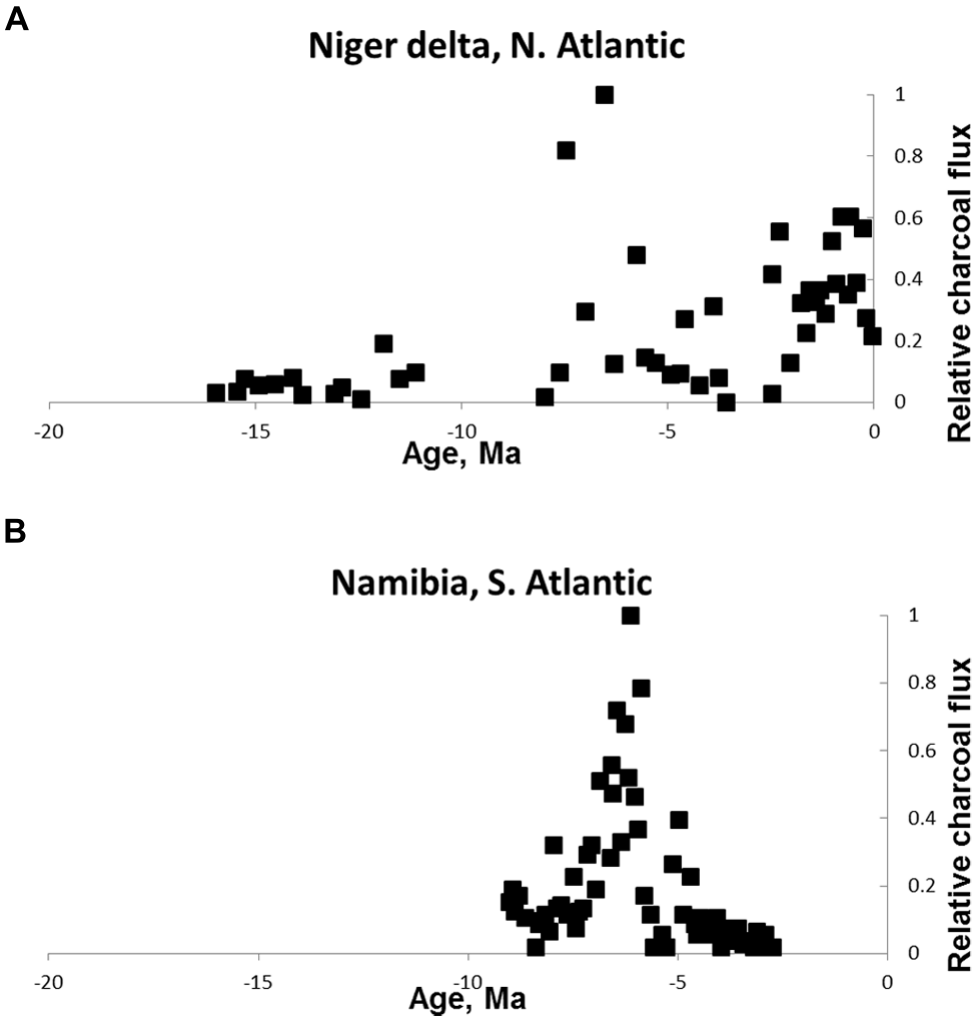
**Neogene charcoal from Atlantic ocean cores. (A)** Niger delta off West Africa (redrawn from Morley and Richards, 1993) **(B)** off the Namibian coast, south–west Africa (redrawn from Hoetzel et al., 2013). The reduction in fire in the Namibian core is attributed to increasing aridity. Values are relativized to the maximum record of the charcoal proxy.

On the other side of the world in the Atlantic Ocean, Morley and Richards (1993) reported changing fire activity associated with changing grass abundance in the Niger Delta off West Africa. Charcoal in the form of charred grass cuticles increased from the late Miocene (~7 Ma) and was associated with a sharp increase in grass pollen indicating the presence of fire-prone savannas (**Figure 3A**). A second site in the south Atlantic was studied by Hoetzel et al. (2013). They explored the role of fire in savanna expansion by studying an ocean core off the Namibian coast. They reported an increase in fire activity from ~7 MA associated with an increase in grass pollen and an increase in carbon derived from C4 carbon sources (**Figure 3B**). The later part of the record shows a decline in fire activity as conditions became too arid to support fuel for fires indicated by increasing pollen from arid-adapted plants characteristic of the dry climate that prevails in the Namib desert of the present day. This interpretation is consistent with present-day fire occurrence with savanna fires common where there is sufficient rainfall to produce continuous fuels but rare or absent in arid regions (e.g., Archibald et al., 2009).

**FIGURE 4:**
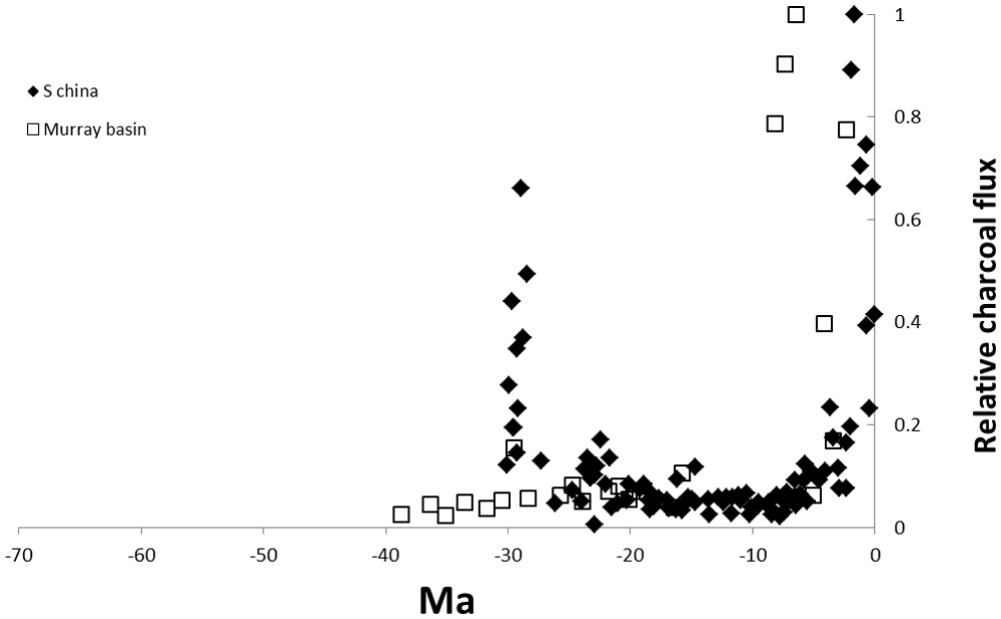
**Cenozoic charcoal records from ocean cores in the South China sea (e.g., Jia et al., 2003) and terrestrial charcoal from the Murray Basin, Australia (e.g., Kershaw et al., 2002).** The time scale is the same as **Figure 2** for comparison of the different locations. Values are relativized to the maximum record of the charcoal proxy.

Finally, on the far side of the world, Jia et al. (2003) reported black carbon fluxes for an ocean drilling site in the South China Sea. The core covers most of the Neogene (from 30 Ma). The earliest part of the record showed significant fire activity followed by a decline with low fire activity until a large increase in black carbon from the Pliocene (~5 Ma; **Figure 4**).

**FIGURE 5:**
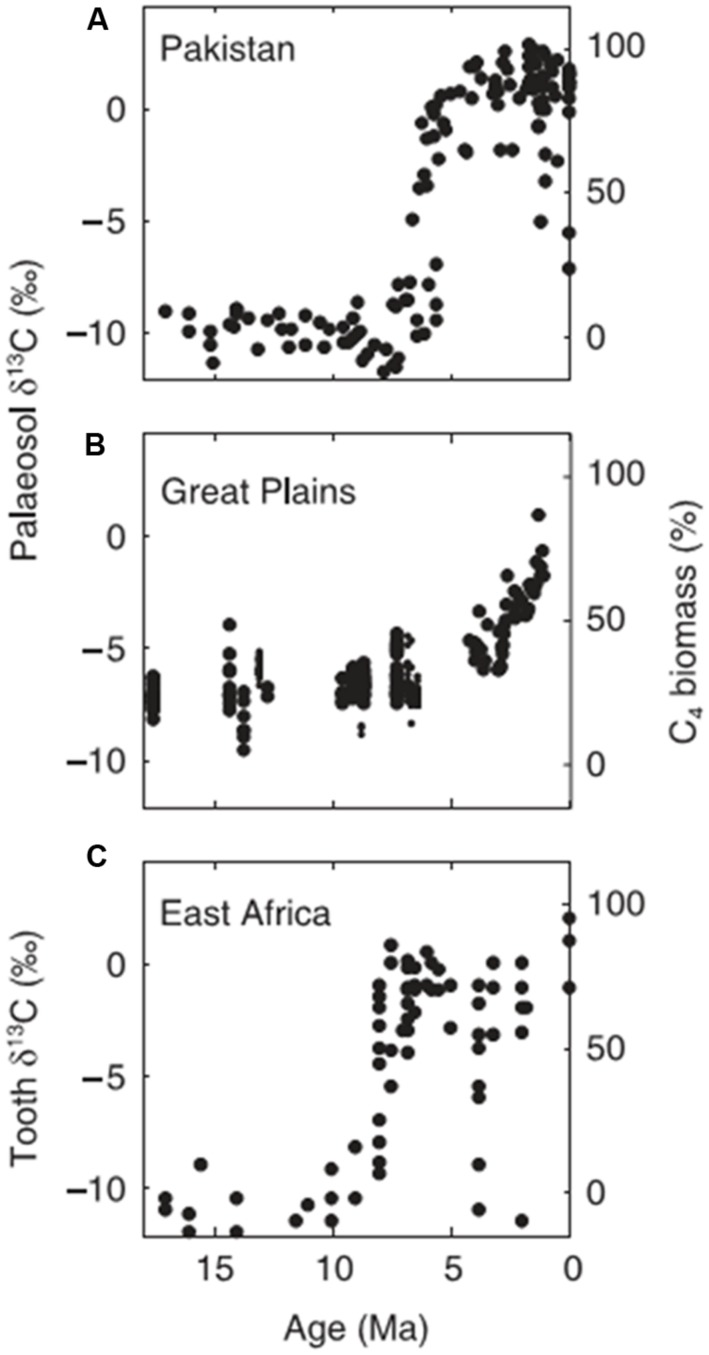
**Examples of the shifts in d^**13**^C associated with the rise of savannas from the Late Miocene derived from palaeosols **(A)**Quade and Cerling (1995), **(B)**Fox and Koch (2003), and **(C)** tooth enamel (Cerling et al., 1997).** Figure from Osborne (2008; with author’s permission).

All these marine charcoal records, from widely separated geographic regions, indicate low but significant fire activity throughout the Cenozoic until the late Miocene or Pliocene (5.3–2.6 Ma) when all show a marked increase in fire activity. In several instances charred grass cuticles were recovered from the cores and the Namibian study also showed changes in the carbon isotopic signal consistent with an increase in C4 grasses (Hoetzel et al., 2013). The geographic source of the charcoal is generally uncertain and cores nearer land may include both fluvial and aeolian sources. Nevertheless, the coincidence of fire activity in these widely separated geographic regions matches well with the rise of the savanna biome from the Late Miocene, as recorded in fossil carbon paleosols and animal teeth (Ségalen et al., 2007 for Africa; Singh et al., 2013 for South Asia; Cerling et al., 1993, 1997; Fox and Koch, 2003 for North America; **Figure 5**). Thus there is general support from charcoal in ocean cores for the hypothesis that increased fire activity was implicated in the spread of the savanna biome in the Neogene (Keeley and Rundel, 2005; Beerling and Osborne, 2006; Osborne, 2008, 2011; Scheiter et al., 2012).

In contrast to the marine record, there seems to be very little fossil evidence for fire in terrestrial studies of the origin and spread of grassy biomes (Strömberg, 2005, 2011). In Eurasia, the initial retreat of forests and their replacement by grasslands has been attributed to increasing aridity from the Miocene (Strömberg, 2011). A well supported example comes from an analysis of numerous Cenozoic pollen cores from central Europe to China (Tang and Ding, 2013). These authors related pollen types to climate, especially precipitation. They showed that temperate deciduous forests were initially widespread in the Eocene but progressively shrank through time and were replaced by arid adapted plants, including C3 grasses forming steppe grasslands, by the late Miocene. The important implication is that the introduction of a novel grass-fuelled fire regime was not the only route for grassland expansion in the Neogene. Aridity in some regions was the major factor in the retreat of Paleogene forests.

### PHYLOGENETIC EVIDENCE

**FIGURE 6:**
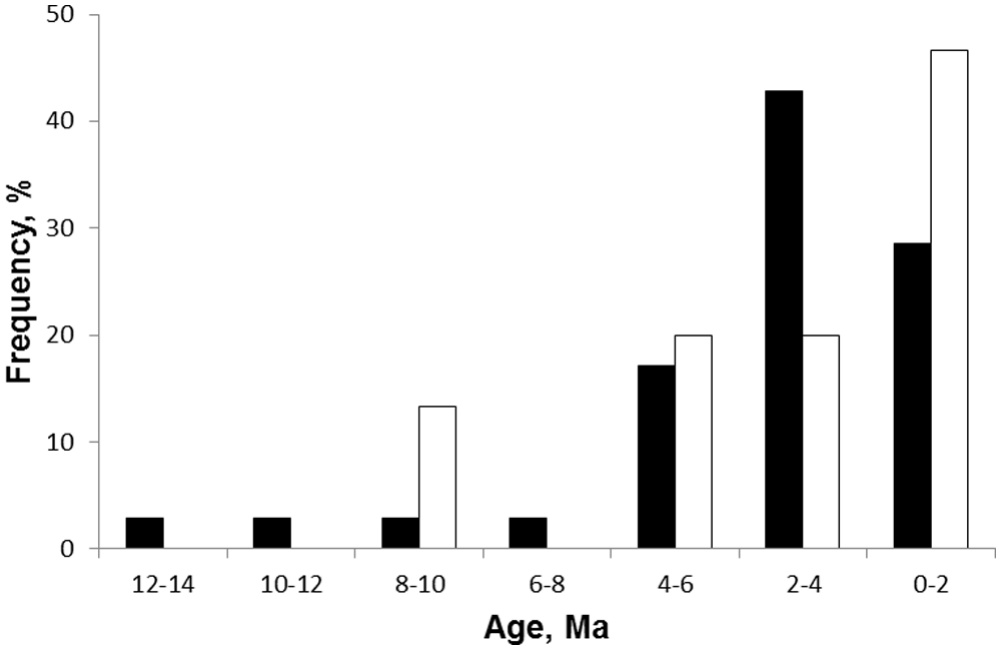
**Frequency distribution of estimated ages from phylogenies of fire adapted woody clades in African (black) and South American (clear) savannas.** The African data is for the age of splits of geoxyles (underground trees) from their sister tree species (from Maurin et al., 2014; *n* = 36). The South American data is for 15 legume lineages in cerrado (from Simon et al., 2009).

The first phylogenetic study tracing the geological history of pyrophytic vegetation was that of Simon et al. (2009) for Brazilian cerrado. This study used dated phylogenies of legume clades to explore the origin of woody species in these high rainfall savannas. The savanna species were all derived from ancestors occurring in closed tropical forests. They diverged from forest ancestors in traits such as thick bark, underground storage organs, and clonal spread considered to be adaptations for frequent grass-fuelled surface fire regimes (Hoffmann et al., 2003; Simon and Pennington, 2012). Molecular dating of the phylogenies indicates that savanna species split from their forest ancestors from ~9 Ma with most lineages diversifying from 4 Ma or less with a median age of 2.3 Ma. This recent origin has been supported by additional studies on clades for which dated phylogenies are known with most cerrado lineages diversifying from 5 Ma or less (Simon and Pennington, 2012; **Figure 6**). Thus fire is directly implicated in the origin of savannas in Brazil and dates of origin are consistent with isotopic evidence for the general rise of savannas (Cerling et al., 1997) and the marine charcoal record of increasing fire activity from other parts of the world.

Recently Maurin et al. (2014) reported a phylogenetic analysis designed to test the dates of origin of fire-dependent savannas in Africa. They focused on “underground trees,” midget trees (<1 m) with close relatives that are tall trees. The underground trees (=geoxyles in the scientific literature) only occur in savannas in seasonally wet regions with frequent fires. The climatic conditions are suitable for forests and, indeed, forest patches are common as alternative biome states in African savanna landscapes (Hirota et al., 2011; Staver et al., 2011). Thus underground trees are indicators of fire dependent savannas occurring in climates that can support forests. Plants with the same geoxylic lifeform are common in Brazilian cerrado but have evolved from different clades indicating convergent evolution in response to similar fire regimes and growing conditions (Maurin et al., 2014). Maurin et al. (2014) used a molecular phylogeny to date divergence times of underground trees from their tall tree relatives as a conservative means of dating the origins of fire dependent savannas. Geoxyles had a median divergence time of 2.28 Ma but with the origins of many taxa dated to within the last 2 Ma indicating a recent expansion into savanna (**Figure 6**). There was a latitudinal gradient in maximum estimated divergence times with oldest dates (Miocene) at low latitudes near the equator and youngest maximum ages in southern subtropical latitudes (Maurin et al., 2014, their **Figure 6**). Thus phylogenetic evidence from both South America and Africa supports fire as a mechanism promoting savanna expansion at the expense of closed forests from the late Miocene continuing into the Plio-Pleistocene. As yet, no terrestrial fossil records have been reported which can test the origin of these savannas and their link to frequent grass-fuelled fires. Indeed well preserved fossils are unlikely in the deeply weathered landscapes characteristic of these high rainfall tropical savannas.

## TEMPERATE FLAMMABLE ECOSYSTEMS

### FOSSIL CHARCOAL

The most intensively studied temperate flammable ecosystems are shrublands in Mediterranean type climates (reviewed by Keeley et al., 2012) and the shrublands and eucalypt woodlands of the southern two thirds of Australia (reviewed by Crisp and Cook, 2013). Herring’s (1985) analysis of ocean drilling cores in the North Pacific included sites close to the Californian coast and therefore adjacent to chaparral. Charcoal fluxes at this site show a similar pattern to those of lower latitudes with low charcoal flux for most of the Cenozoic but a rapid increase from ~5 Ma, somewhat later than tropical cores (**Figure 2**). There seem to have been no comparable studies exploring fire activity from charcoal in Neogene marine sediments in the southern hemisphere. Kershaw et al. (2002) summarized the results of two terrestrial cores in southern Australia which sampled mid-to late Cenozoic material. In the Murray Basin site, no charcoal was recorded in the Eocene and charcoal remained negligible until the late Miocene (**Figure 4**). The Latrobe valley site also showed negligible charcoal deposits in older deposits but charcoal began increasing from the mid-Miocene with high charcoal content from the late Miocene (Kershaw et al., 2002). In south-western Australia, varved lake sediments from the Pliocene (~3 Ma) have revealed recurrent fires at high frequencies in a mosaic of heathlands and forests (*Nothofagus, Araucaria*; Atahan et al., 2004; Dodson et al., 2005). The rainforest elements no longer exist in Western Australia. They are fire-sensitive and largely restricted to fire refugia in eastern and northern Australia (Bowman, 2000).

In the Cape region of south–west Africa, no continuous records of Cenozoic deposits have been analyzed for charcoal. Burnt logs of *Podocarpus*, typically a forest tree, and charcoal rich layers have been reported from the West Coast in sediments thought to be of mid-Miocene age (Roberts et al., 2013). Evidence of fire has also been reported for Pliocene (5 Ma) deposits of Langebaanweg. The data have not been critically examined for evidence of repeated fires (a fire regime) versus rare catastrophic fires. The vegetation at Langebaanweg was a mosaic of woodland, C3 grassland and some fynbos elements and supported a rich megafauna including giraffids, equids, suids (pigs), bovids, rhinocerotids, hippopotamids, and elephantids (Franz-Odendaal et al., 2002). This Pliocene ecosystem contrasts strikingly with the present-day low pyrophytic fynbos shrublands supporting a sparse modern fauna of small-bodied antelope (Klein et al., 2007). The implication is a massive faunal and vegetation turnover in the last 5 Ma on these coastal lowlands.

### PHYLOGENETIC EVIDENCE

In contrast to the above fossil evidence for a mid to late Miocene rise in fire activity, the phylogenetic evidence for Cape and Australian pyrophytic shrublands and woodlands suggests continued fire activity for the entire Cenozoic (Keeley et al., 2012; Crisp and Cook, 2013). Lineages that are restricted to pyrophytic ecosystems and which possess fire adaptations such as serotiny and fire-stimulated flowering, were diversifying in the Paleogene and later. Phylogenetic studies of clades that currently occur in temperate pyrophytic woody ecosystems have revealed the considerable antiquity of fire adaptive traits. He et al. (2011) traced the origin of *Banksia*, a genus characteristic of Australian flammable shrublands today, to the mid-Cretaceous. Serotiny is a fire-adaptive trait in which seeds are retained in insulated cones or follicles until released en masse after fire. The *Banksia* phylogeny indicated that serotiny was present from the first appearance of the genus at ~60.8 Ma. Lamont and He (2012) explored the origins of pyrophytic vs. rainforest Proteaceae, including both Australian and African clades, and traced the origin of the pyrophytes to the mid-Cretaceous. Crisp et al. (2011) explored the origins of the distinctive epicormic strands in eucalypts as a marker of a fire adaptation unique to the clade and found Paleocene (60 Ma) origins based on a dated phylogenies. In the Cape region, Bytebier et al. (2011) reported phylogenetic evidence for the origin of fire stimulated flowering in fynbos orchids from the mid-Miocene (~15 Ma). Crisp and Cook (2013) reviewed the phylogenetic evidence for the evolution of Australian biomes including the continental dominance of contemporary pyrophytic vegetation. They concluded that there was a long history of fire adapted woody species from at least the Paleocene with a major expansion of flammable pyrophytic vegetation (=sclerophyll vegetation) at the expense of closed forests from the Miocene. This is consistent with the analysis of Keeley et al. (2012) in indicating significant fire activity in flammable woody vegetation more or less continuously throughout the Cenozoic. This view contrasts with the apparent lack of fossil evidence for fire activity from the Eocene until the mid-Miocene (e.g., Kershaw et al., 2002). Keeley et al. (2012) discuss possible reasons for the poor match between fossils and phylogenies and note that much of the burning may have taken place in drier upland vegetation with low probabilities of fossil formation.

In pyrophytic woody vegetation of both the Cape region of Africa and Australia, there are ancient lineages that occur in fire prone vegetation dating to the early Cenozoic or even the Cretaceous (reviewed in Linder, 2005, Verboom et al., 2009 for the Cape; Crisp and Cook, 2013 for Australia). However, in both regions there is also evidence for lineages in flammable shrublands that radiated from the late Miocene. An early origin of some flammable lineages is to be expected if, as suggested by Bond and Scott (2010), the spread of Cretaceous angiosperms was promoted by fire. Paleocene fires from fossil charcoal studies have been reported as common and essentially unchanged from the fiery world of the Cretaceous (Collinson et al., 2007; Belcher et al., 2013). This all changed from the Eocene (55 Ma) according to the general fossil record with the development of very extensive closed forests which would have shaded out fire-adapted flammable plants (Willis and McElwain, 2002). Nevertheless, fossil evidence for open, non-forested ecosystems, though rare, does exist in the fossil record of the Eocene (Keeley et al., 2012).

## EDAPHIC GHETTOS

One of the puzzling features of C4 grass evolution is the long time lag between their origins in the Oligocene (30 Ma+; Christin et al., 2008; or earlier; Urban et al., 2010) and their much later Miocene–Pliocene assembly and spread as the savanna biome (7–8 Ma; Cerling et al., 1997). Where were they in the 20+ million years before savannas become visible in the fossil record? A similar question can be asked for flammable shrublands and related woody vegetation which had fiery beginnings in the Cretaceous through to the Paleocene but then all but disappeared for 40+ million years until their renaissance in the last 10 Ma.

Fire is not the only factor accounting for low open ecosystems in climates that also support closed forests. Extreme soils, ‘edaphic ghettos’ where trees do not grow, provide habitat for open ecosystems. Noss (2012) has reviewed the diverse nature of soil types hostile for tree growth in the south-eastern USA where edaphic grasslands contribute to the very high diversity of the region. Grassland refugia include soils too shallow to support trees or with claypan layers or that are seasonally waterlogged thus restricting root growth. In shallow rocky soils, such as those of the mountains in the Southwest Cape of South Africa, tree growth is also restricted by shallow rooting depth. Heavy clay soils (vertisols) exclude trees, for example in parts of the southern USA (Noss, 2012) and Northern Australia (but less so in Africa). Forests also tend to occur on more nutrient rich soils than open flammable ecosystems and nutrient constraints on tree growth are often cited to explain the presence of non-forested ecosystems in wet, warm climates. However, an analysis of nutrient stocks required to build a forest (Bond, 2010) found no evidence for absolute nutrient constraints on forest formation except on very shallow soils where physical constraints on rooting depth would also limit forest development through both nutrient limitation and soil aridity. Edaphic ghettoes may have been key habitats supporting shade-intolerant pyrophytic plants when environmental conditions generally favored closed forests. In the Cape region, for example, it has been suggested that fynbos shrublands persisted in otherwise forested landscapes throughout the Cenozoic on shallow, rocky, nutrient-poor soils of the Cape mountains (Linder, 2003; Verboom et al., 2009; Hoffmann et al., in press). However, as Keeley et al. (2012) have argued, pyrophytic vegetation may also have been invisible in the fossil record because of taphonomic biases against fossil formation in drier uplands where fires were most likely to burn.

If fire activity showed a widespread increase in the late Neogene, as suggested by the charcoal record (**Figures 2**–**4**), then there should be phylogenetic markers indicating the expansion of flammable shrublands into habitats previously occupied by closed fire-resistant forests. As discussed earlier, phylogenetic signals of changing biome extent are subtle and harder to detect than the origin of a new biome. If habitats opened up by fire exposed plants to the same climate and soil conditions as in their ancestral refugium, there would be little selection pressure for diversification. Indeed speciation rates might decline as previously fragmented patches coalesce promoting gene flow (Linder, 2008; Bouchenak-Khelladi et al., 2014). If, however, new habitats were opened up as forests retreated, with different soils and climate conditions, then speciation would be expected to increase so that the newly exposed habitat would support more recently derived lineages. This kind of reasoning has been applied to the Cape flora. Fossil evidence suggests lowland vegetation along the coastal regions was forest and woodland or at least a mosaic of forest and fynbos until the mid-Miocene (Coetzee and Rogers, 1982; Coetzee and Muller, 1984; Carr et al., 2010; Roberts et al., 2011, 2013). The lowland forests retreated and were replaced by flammable shrublands though dates are uncertain (see e.g., Carr et al., 2010; Roberts et al., 2013). The change from lowland forests and woodlands to pyrophytic fynbos shrublands is usually attributed to climate change and especially the appearance of winter-wet/summer-dry Mediterranean climate (e.g., Goldblatt and Manning, 2002). Another suggestion is that new geological substrates were exposed linked to Late Cenozoic geomorphological changes (Cowling et al., 2009). However, if fires became more frequent and more severe as a result of a more predictable, intense dry season, then forests would be likely to retreat exposing new land surfaces for fynbos colonization.

Hoffmann et al. (in press) analyzed speciation in 11 Cape clades in relation to climate (aridity, seasonality) and geological substrate. They found that the upland flora on oligotrophic quartzites was ancient and generally associated with aseasonal mesic climates. However, the lowland flora shows evidence for rapid radiation of clades in response to the development of seasonal climates (and not aridity *per se*) and exposure of new soil types dating from the Late Miocene (~7–8 Ma). This pattern is consistent with increased fire activity from the Late Miocene promoting the spread of flammable shrublands from their mountain refugia into the Cape lowlands replacing lowland forests and woodlands. The relative importance of direct effects of rainfall seasonality on trees versus the indirect effects of fire on forest distribution is poorly understood.

## SUMMARY OF FIRE IN THE CENOZOIC

Contrary to the terrestrial fossil record, fires continued to burn throughout the Cenozoic based on phylogenetic evidence from Mediterranean shrublands and sclerophyll ecosystems in Australia. Fires probably also burnt patches of edaphically maintained grasslands, including those with a significant C4 component. Both the marine charcoal data and the phylogenetic evidence indicate increasing fire activity from the middle and especially the late Miocene. This is especially true for savannas of the tropics and subtropics as emphasized by Keeley and Rundel (2005). The late Cenozoic increase in fire activity occurred in widely separated geographic regions. It occurred in savannas with grassy fuels but also in shrublands and woodlands with woody fuels. The apparent synchrony of increased fire activity in low latitudes with the appearance of traits adaptive to frequent surface fire regimes is consistent with hypotheses invoking fire as a key driver of savanna expansion (Keeley and Rundel, 2005; Beerling and Osborne, 2006; Scheiter et al., 2012). Our world is not a low fire world as suggested by the inertinite record and scarcity of terrestrial charcoal over much of the Cenozoic. Fires became much more frequent from ~10 Ma. Contemporary flammable biomes of the world began to spread from this time. In the case of savannas, and related C4 dominated grasslands, this process continued with new areas being converted as recently as the last 2–3 Ma.

## CAUSES OF INCREASED FIRE ACTIVITY

There has been intense interest in the causes of the rapid rise of the savanna biome from ~8 Ma (Ehleringer et al., 1997; Keeley and Rundel, 2005; Osborne, 2008, 2011; Edwards et al., 2010; Scheiter et al., 2012). This review of fossil and phylogenetic data supports the hypothesis that increased fire activity was a major factor promoting the spread of C4 grasslands at the expense of forests. To the savannas, we can also add southern flammable shrublands and associated Australian woodlands. The problem, now, is how to explain the surge of fire activity from the late Miocene in so many different geographic regions.

Several studies have attributed variation in fire activity in different geological periods to changes in atmospheric oxygen. There are no widely accepted direct proxies for ancient oxygen so that its changing contribution to the atmosphere has to be estimated from geochemical models (Berner, 2006, 2009; Bergman et al., 2004; Lenton, 2013). These models vary in their reconstruction of atmospheric oxygen in the Cenozoic but two of the most widely cited studies simulate declining oxygen from high levels in the Cretaceous to present atmospheric levels of 20.9% (Bergman et al., 2004; Berner, 2009). Thus, if the models are to be believed, the late Neogene increase in fire activity is not linked to increasing oxygen.

Climate change is by far the most common explanation for vegetation change in the past. For example, the expansion of Cape fynbos at the expense of forests has been attributed to the onset of Mediterranean climates with their wet winters and summer droughts (Goldblatt and Manning, 2002). So can increased fire activity from the late Miocene be attributed to changing climates? The general increase in aridity through the Neogene associated with cooling (Pagani et al., 1999; Zachos et al., 2001) would not promote fires if the vegetation became too sparse to support fires (see e.g., Krawchuk and Moritz, 2011; Hoetzel et al., 2013). Keeley and Rundel (2005) argued that the development of monsoonal climates with wet summers and dry winters promoted frequent fires which triggered the expansion of savannas. A reliable wet season promotes rapid plant growth (fuel) while a long dry season creates dry fuels conducive to burning. A long dry season emerged as a major climate predictor, after annual rainfall, in an analysis of determinants of savanna distribution on the southern continents (Lehmann et al., 2011). The wet–dry climate is not essential for savannas – some, such as the pine savannas of the south-eastern USA, occur in non-seasonal rainfall regions (Noss, 2012).

Keeley and Rundel (2005) cited the emergence of the Asian monsoon in the Late Miocene as evidence for a change towards the wet/dry cycle needed to promote savannas. However, diverse lines of evidence suggest that the Asian monsoon began to develop earlier in the Miocene (Sun and Wang, 2005; Osborne, 2011). The monsoonal climate hypothesis would also require near simultaneous onset of seasonality in diverse geographic regions in different climatic settings. There is, indeed, growing evidence for a global monsoon with teleconnections across continents that would influence rainfall seasonality in widely divergent areas (Trenberth et al., 2000; Wang et al., 2012). The paleo-history of this global climate phenomenon is a subject of current research (Huber and Goldner, 2012) but the phenomenon is still too poorly known to link to the emergence of high fire activity from the late Miocene. The monsoon is traditionally viewed as a low latitude phenomenon while the evidence reported here indicates that the surge of fire activity in the late Neogene also occurred at mid-latitudes. In the Cape region of South Africa, the onset of Mediterranean-type climates is usually attributed to the development of the cold Benguela current (e.g., Dupont et al., 2011; Hoffmann et al., in press). Thus the seasonality hypothesis is a reasonable one from our current knowledge of controls on fire regimes (e.g., Krawchuk and Moritz, 2011) but our understanding of changes in global climates that might have caused a global change in fire regimes is still too rudimentary to provide a strong case for climate as the main cause.

Beerling and Osborne (2006) suggested that savanna fires would trigger positive feedbacks on the atmosphere causing climates to change in ways that would further promote the spread of savannas. An analogy would be a room full of explosive gases just waiting for a match. The problem with the hypothesis is explaining the apparent synchrony of increased fire in diverse geographic regions some of which are isolated from each other by ocean barriers (e.g., **Figure 6** for African and South American savannas). The analogy would be arsonists carefully distributed in all suitable regions and all striking their matches at the same time.

Changing atmospheric CO_2_ has emerged as a potential global driver of vegetation change. CO_2_ is well mixed in the atmosphere so that changes in CO_2_ are experienced globally. Ehleringer et al. (1997) argued that C4 grasses would have gained a photosynthetic advantage over their C3 predecessors when atmospheric CO_2_ fell below 500 ppm, beginning in the tropics in the late Miocene. Subsequent studies of paleo-atmospheres have shown that CO_2_ first fell below this threshold in the Oligocene 20+ millions of years before the spread of savannas (Pagani et al., 1999; Beerling and Royer, 2011; Zhang et al., 2013) leading to the rejection of the CO_2_ hypothesis for savanna spread. However, a recent study using novel marine algal proxies indicates a steep decline in CO_2_ from ~7 Ma (Bolton and Stoll, 2013) consistent with the original idea. The photosynthetic mechanism proposed by Ehleringer et al. (1997) would matter most for plants with the same growth form such as C3 versus C4 grasses. However changing CO_2_ can also alter the balance between herbaceous and woody plants as a result of their divergent responses to fire (Bond and Midgley, 2000; Bond et al., 2003b). Under low CO_2_ conditions, grasses can recover from a fire with enough biomass to burn again much more rapidly than woody plants can recover sufficient above and below ground biomass to resist a follow-up fire (Bond et al., 2003b). The consequence is that trees damaged by fire will recover more slowly, and forest saplings colonizing flammable communities will grow more slowly leading to a retreat of forest boundaries. Woody plant growth rates are a key feature of current conceptual models for the stability of forest/savanna boundaries (Hoffmann et al., 2012) but CO_2_ effects have yet to be quantified. Both simulation studies and experiments have shown strong CO_2_ effects on tree populations with elimination of trees in savannas at CO_2_ levels characteristic of the last glacial if fires continued to burn (Bond et al., 2003b; Kgope et al., 2010; Bond and Midgley, 2012 and see Quirk et al., 2013 for CO_2_ /drought interactions). The minimum threshold at which a CO_2_ effect on trees would be expressed as a retreat of the forest boundary is not yet known. The few experimental studies suggest it is lower than the 500 ppm threshold for C4 versus C3 photosynthetic advantage (Kgope et al., 2010; Quirk et al., 2013). An exploration of woody plant response to low CO_2_ as the key to fire-driven savanna expansion might be warranted, especially if CO_2_ concentrations showed a declining trend (below 500 ppm) from the late Miocene. Uncertainties over the accuracy of CO_2_ proxies limit paleoecological tests (Bolton and Stoll, 2013; Zhang et al., 2013). Inverse modeling of CO_2_ over the last 20 Ma based on isotopic records of ocean temperatures calculated CO_2_ trending below 400 ppm from ~12 Ma, below 350 ppm from ~8 Ma and below 300 ppm from ~3 Ma (van de Wal et al., 2011).

## CONCLUSION

This review of fossil and phylogenetic evidence of fire in the Cenozoic points to a long period of relatively low fire activity from the Eocene (starting 55 Ma) followed by a surge of fire activity within the last 10 million years. There is a puzzling degree of synchrony in the late onset of fire activity in different flammable ecosystems and in widely separated geographic regions. Terrestrial fossil records suitable for exploring Cenozoic fires are few and far between. Marine charcoal records are likely to be particularly useful in filling in the large gaps in the Cenozoic fire record. Phylogenetic analyzes have emerged as a very useful supplement tracing the history of pyrophytic lineages and at this stage suggest a disconnect between terrestrial records of fire and the origin and proliferation of fire traits. However, ecological interpretation can be difficult for both fossil and phylogenetic data. For the latter more thought needs to be given for how diversification patterns in phylogenies can be linked to changes in the extent of the habitat which generates the novel selective environments. Explanations for the changing importance of fire in the Cenozoic expose our limited understanding of the causes of fire in deep time. None of the usual suspects (evolution of new plants and new fuels, changing oxygen, changing climate) seem plausible. The plants that built new fire-dependent biomes were present many millions of years before they began to expand under increased fire activity. Fire activity increased when models predict that oxygen was declining. Regional climate change is not a sufficient explanation for the expansion of fire-dependent ecosystems when the phenomenon appears to be global. I hope the omissions and commissions of this review will help stimulate further work on the intriguing history of fire and pyrophytic vegetation in the Cenozoic.

## Conflict of Interest Statement

The author declares that the research was conducted in the absence of any commercial or financial relationships that could be construed as a potential conflict of interest.
